# Supplementary material for: Triple-Isotope Tracing for Pathway Discernment of NMN-Induced NAD+ Biosynthesis in Whole Mice
Source: Int J Mol Sci. 2023 Jul 5;24(13):11114. doi: 10.3390/ijms241311114 (PMC10342116; doi:10.3390/ijms241311114)
Supplement: Supplementary file 1 [file ijms-24-11114-s001.zip › ijms-2469087-supplementary.pdf]

Preparation of ¹³C-labeled ribofuranose tetraacetate

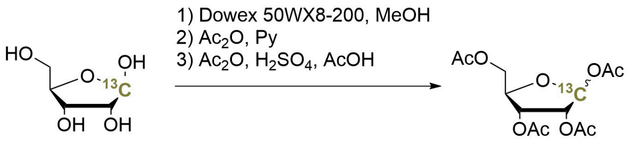

Preparation of ¹⁸O-labeled nicotinamide

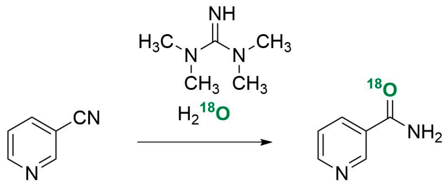

Preparation of isotope-labeled NMN

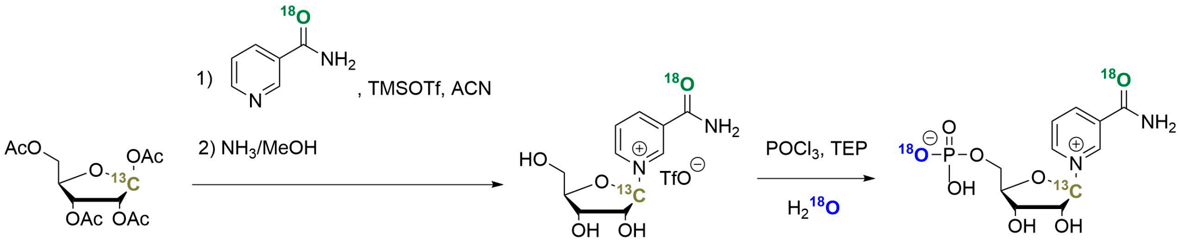

Scheme S1. Synthesis of [^{18}O-phosphoryl-^{18}O-carbonyl-^{13}C-1-ribosyl] NMN.
